# Ambient Benzo[a]pyrene’s Effect on Kinetic Modulation of Amyloid Beta Peptide Aggregation: A Tentative Association between Ultrafine Particulate Matter and Alzheimer’s Disease

**DOI:** 10.3390/toxics10120786

**Published:** 2022-12-14

**Authors:** Samal Kaumbekova, Mehdi Amouei Torkmahalleh, Dhawal Shah

**Affiliations:** 1 Department of Chemical and Materials Engineering, School of Engineering and Digital Sciences, Nazarbayev University, Kabanbay Batyr 53, Astana 010000, Kazakhstan; 2 Division of Environmental and Occupational Health Sciences, School of Public Health, University of Illinois at Chicago, Chicago, IL 60612, USA

**Keywords:** environmental toxicology, benzo[a]pyrene, molecular dynamics simulations, Alzheimer’s disease, amyloid peptide aggregation, oligomerization

## Abstract

Long-time exposure to ambient ultrafine particles is associated with an increased risk of neurodegenerative diseases such as Alzheimer’s disease (AD), which is triggered by the aggregation of Aβ peptide monomers into toxic oligomers. Among different ultrafine air pollutants, polycyclic aromatic hydrocarbons (PAHs) are known to have a negative neural impact; however, the impact mechanism remains obscure. We herein examined the effect of Benzo[a]Pyrene (B[a]P), one of the typical PAHs on Aβ_42_ oligomerization using all-atom molecular dynamics simulations. In particular, the simulations were performed using four molecules of Aβ_42_ in the presence of 5.00 mM, 12.5 mM, and 50.0 mM of B[a]P. The results revealed strong hydrophobic interactions between Aβ_42_ peptides and B[a]P, which in turn resulted in increased interpeptide electrostatic interactions. Furthermore, 5.00 mM of B[a]P accelerated the kinetics of the formation of peptide tetramer by 30%, and stabilized C-terminus in Aβ_42_ peptides, suggesting consequent progression of AD in the presence of 5.00 mM B[a]P. In contrast, 12.5 mM and 50.0 mM of B[a]P decreased interpeptide interactions and H-bonding due to the aggregation of numerous B[a]P clusters with the peptides, suppressing oligomerization kinetics of Aβ_42_ peptides by 13% and 167%, respectively. While the study elucidates the effect of small environmental hydrophobic molecules on the formation of Aβ oligomers, the impact of ambient ultrafine particles on AD in the complex composition of the environmental realm requires further systematic delving into the field.

## 1. Introduction

Environmental air pollutants are known to have a negative neural impact [1], with the effect ranging from an increased risk of autism spectrum disorders, brain volumetric changes, brain inflammation, cerebrovascular and neuropsychiatric disorders, and the hallmarks of Alzheimer’s disease (AD) [2,3,4,5,6]. The concern about the development of AD-like neurodegenerative diseases increases with the growth of the elderly population globally. AD is associated with the development of amyloid plaques due to the aggregation of amyloid beta (Aβ) peptide monomers into soluble oligomers, and the formation of neurofibrillary tangles in the human brain [7]. The pathogenesis of AD also implicates genetic and environmental factors [8].

Among different air pollutants, Polycyclic Aromatic Hydrocarbons (PAHs) are associated with an increased risk for neurodegeneration [9]. Benzo[a]pyrene (B[a]P, C_20_H_12_) is a typical PAH, with five aromatic rings in its structure. B[a]P is usually generated from incomplete combustion of organic material, motor-vehicle exhaust, and from cooking and smoking. According to source apportionment measurements, the average concentration of B[a]P in ambient air might reach up to 1.94 ng/m^3^ in highly industrialized regions of Italy [10]. Long-term monitoring analysis of ambient air in the Czech Republic revealed high levels of B[a]P (up to 7.7 ng/m^3^) in industrialized regions, in addition to the elevated concentrations of B[a]P (up to 13.6 ng/m^3^) observed during the winter season due to the household and local heating [11]. The mean values of average annual concentrations of B[a]P and a total of 24 PAHs in the ultrafine particles in different regions of China measured from October 2012 to September 2013 were 5.02 ng/m^3^ and 53.5 ng/m^3^, respectively [12]. Hydrophobic ultrafine molecules, such as PAHs, can diffuse through cell membranes [13] and cross the blood–brain barrier [14]. The concentration of PAHs in the blood of children in Nigeria varied from 535 to 708 µg/L [15]. The effect of B[a]P on the neurobehavioral functions of coke oven workers was recently studied in Taiyuan, China [16]. The concentrations of B[a]P observed in the coke oven’s bottom, side, and top regions were 19.5 ± 13.2, 185.9 ± 38.6, and 1623.5 ± 435.8 ng/m^3^, respectively. In addition, the authors evaluated the neurobehavioral function of coke oven workers and observed a statistical decline in the exposed group.

The formation of soluble aggregates of Aβ peptides in the human cerebrospinal fluid is related to the progression of AD via synaptic and neuronal loss [17]. The aggregation of Aβ peptides can be observed in various brain regions at distinct stages of AD [18]. According to the amyloid cascade hypothesis, the aggregation of Aβ peptides into β—sheets via fibrillization leads to the formation of amyloid plaques in different areas of the brain [19]. Soluble Aβ peptide oligomers are considered the most toxic form of amyloid aggregates associated with neuronal damage [20]. According to clinical studies, the concentration of Aβ oligomers may reach up to 2647 fg/mL in the human cerebral fluid (CSF) of AD patients with moderate dementia [21]. According to cell binding and toxicity models in cortical neuronal cultures, tetramers possess higher neurotoxicity among different Aβ oligomers with lower molecular weight [22]. Amyloid fibrils and aggregates are formed more rapidly from the Aβ_42_ peptide variant, with 42 aminoacids in its structure among different Aβ peptide isoforms with 39 to 43 aminoacids [23]. While the first 16 aminoacid residues of the N-terminus of Aβ_42_ are hydrophilic, the remaining region of the peptide is hydrophobic, including the central region (Aβ_12–23_) and C-terminus (Aβ_30–42_) [24,25].

A recent molecular dynamics (MD) study showed that B[a]P molecule decreased the amount of helices, promoting the formation of β-sheets and β-bridges in the secondary structure of Aβ_42_ peptide monomer [26]. Furthermore, in vitro experiments on the effect of cigarette smoke components on the structure and aggregation of Aβ peptides showed that PAHs increased the aggregation kinetics of Aβ peptides [27]. In particular, the presence of B[a]P decreased the aggregation halftime from 6.5 ± 0.9 h (for 10–20 µM of Aβ_40_ dissolved in 20 mM of buffer) to 5.1 ± 0.7 h (Aβ dissolved in 100–200 µM of B[a]P) [27]. According to Gao et al. [28,29], chronic exposure to B[a]P increases Aβ_42_ concentrations in the brain of the zebrafishes. In addition, the study showed that cognitive, memory, and locomotor activities of the fish decreased after the B[a]P exposure. Moreover, Liu et al. [30] observed elevated concentrations of Aβ monomers and oligomers, as well as the formation of Aβ plaques and Aβ fibrils in the brain of mice due to the exposure to B[a]P.

While in vivo and in vitro experiments showed enhanced aggregation of Aβ peptides and the formation of amyloid plaques in the presence of B[a]P, the molecular interactions between Aβ peptides and the pollutant are obscure. In this study, we performed a molecular dynamics study aimed to investigate the effect of B[a]P on the structure of Aβ_42_ peptide and oligomerization kinetics of four Aβ_42_ peptide monomers. Furthermore, to investigate the impact of varying concentrations of B[a]P on the oligomerization of Aβ_42_ peptides, the molecular dynamics simulations, each of 500 ns, were performed in the presence of different concentrations of B[a]P molecules. Moreover, to get statistically relevant results within reasonable computational time in the MD study, the concentrations of the peptides and B[a]P molecules inserted in the simulation box were higher than what would be found in in vitro and in vivo studies [27,28]. In particular, to investigate the impact of B[a]P, we performed simulations in the presence of 4 B[a]P molecules (5 mM), keeping the 1:1 ratio between the peptide and air pollutant, as was used in our previous study, where we simulated systems with 1 monomer and 1 B[a]P molecule [26]. The highest concentration of B[a]P used in the simulations was 50 mM, with the 10:1 ratio between B[a]P and peptides, as was previously used in the experimental study performed by Wallin et al. [27]. The choice of 12.5 mM was taken as an intermediate concentration between the two values. Considering that the simulation box is a simple model of a physical system, the aggregation of peptides occurs faster in the MD simulations, while the results of the MD studies represent the general trend that would be expected to be found in an actual system.

## 2. Methodology

Atomistic molecular dynamics simulations were performed via Gromacs 2019.6 software [31]. Gromos54a7 forcefield parameters were chosen based on the literature validations [32] and our previous study with the validation of the density of B[a]P at 20 °C [26]. The molecular dynamics simulations were performed in a simulation box with the dimensions of 11 × 11 × 11 nm^3^. Four Aβ_42_ monomers (PDB ID: 1Z0Q, with a total charge of −2 on each peptide) were inserted in a simulation box, keeping the concentration of peptides at 5.00 mM. The system was solvated via SPC water model. 0.15 M of NaCl salt was added as a buffer environment. The topology parameters and optimized geometry of B[a]P molecule (C_20_H_12_) were taken from the Automated Topology Builder (ATB, Version 3.0) [33]. The concentration of B[a]P varied from 0 mM and 5.00 mM, 12.5 mM, and 50.0 mM, corresponding to 4, 10, and 40 molecules of B[a]P. Due to the limitations of the simulation time and box size, the concentrations of Aβ_42_ peptide and B[a]P molecules in the simulated systems were comparatively higher than the molecular concentrations normally found in the human blood [15]. However, the relative amount of B[a]P to Aβ, which is 10:1, is similar to those used in the in vitro experiments [27]. The number of molecules used in the simulated systems is shown in Table 1.

Energy minimization was performed with the steepest descent algorithm, applying periodic boundary conditions in all directions, and setting the maximum force for convergence of 100 kJ mol^−1^ nm^−1^. After that, NVT—equilibration step with H-bonds constraints was performed for 25 ps with a time step of 0.5 fs. Next, the NPT—equilibration step with all-bonds constraints was performed for 100 ps with a time step of 2 fs, at reference pressure of 1 bar. LINCS (LINear Constraint Solver) constraint algorithm [34] with LINCS iterations of 1 was applied at reference temperature of 298 K. Short-range cutoff distance of 1 nm was applied for both electrostatics and van der Waals interactions with Verlet cut-off scheme algorithm. PME (Particle-mesh Ewald) was set for long-range electrostatic interactions. Molecular dynamics simulations were performed for 500 ns with a time step of 2 fs, considering reaching an equilibrium within a simulated time in all systems under the study, as discussed below. The output coordinates, velocities, and energies were saved every 4 ns.

The oligomerization (and kinetics) of four Aβ_42_ peptides were investigated by using cluster and intermolecular distance analyses. The intermolecular distances were calculated based on the center of masses (COM) of the residues and taking the average values between the distances between COM of peptides 1 and 2, peptides 1 and 3, peptides 1 and 4, etc. The formation of the clusters was studied for the defined groups of the residues, “four peptides” and “peptides and B[a]P”, to analyze the formation of the interpeptide and peptide-B[a]P clusters, respectively. The cluster analyses were performed setting 0.35 nm as a maximum distance parameter between the center of masses of the residues within the defined group to be defined as a cluster. Time-evolution of solvent accessible surface area (SASA), radius of gyration (RoG), and secondary structure analyses were performed to investigate the deviations in the structure of the peptides within 500 ns of the simulations. The (radial distribution function) rdf analysis was performed to investigate the probability of finding “peptides” and “B[a]P” residues from “peptides” residues using the COM of the residues. H-bonds analyses, root-mean square fluctuations (RMSF), and (rdf) analyses were performed for the last 30 ns of the simulations to investigate the structure of peptides and intermolecular interactions in the end of the simulations. In addition, visual molecular dynamics (VMD) software [35] was used to visualize the systems under the study. Finally, the non-bonded intermolecular interactions were studied for the last 10 ns of the MD simulations. In particular, the short-range (SR) interactions with the cut-off distance of 1 nm, and long-range (LR) interaction energies were calculated separately. In addition, the Lennard-Jones (LJ) potential and Coulombic (Coul) potential energies were quantified, indicating the repulsion-dispersion and electrostatics interactions, respectively.

Finally, an additional short replica of each system was simulated independently for 250 ns, starting from the randomly inserted molecules in the simulation box. The results of the simulations were validated via interpeptide distance and SASA analyses.

## 3. Results and Discussion

**Figure 1:**
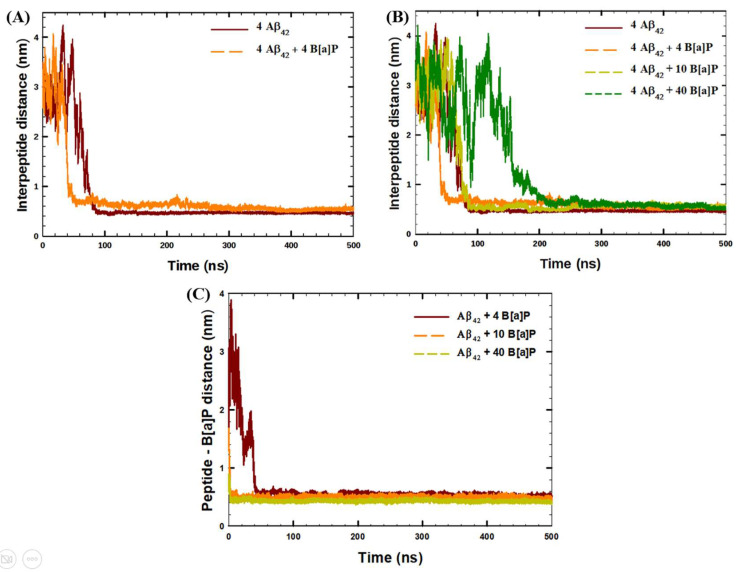
Time-evolution of (**A**,**B**) average interpeptide distances during 500 ns of the simulation, (**C**) average distances between Aβ_42_ peptides and B[a]P molecules during 500 ns of the simulation.

The kinetics of the tetramerization of Aβ_42_ peptides was initially studied in the absence of B[a]P and in the presence of 4 B[a]P molecules via interpeptide distance analysis (Figure 1A). Similarly, the effect of B[a]P concentration on the oligomerization was investigated by interpeptide distance analysis (Figure 1B), followed by the peptide—B[a]P distance analysis (Figure 1C).

The analysis of the interpeptide distances showed that in the absence of B[a]P molecules, four Aβ_42_ peptides aggregated within the simulated time; in particular, within 100 ns of the simulation (Figure 1A). A different trend was observed by Brown et al. [36], wherein the tetramerization of Aβ_42_ was seen within 250 ns of the simulation. The difference in the tetramerization time could be the usage of older forcefield parameters (GROMOS96 53A6), higher temperature of simulations (310 K), and large simulation box (with dimensions of 12.7 nm) by Brown and Bevan [36]. According to Figure 1A, the presence of 4 B[a]P molecules significantly decreased the time required for the oligomerization. The peptides aggregated within 50 ns of the simulation, which was the fastest aggregation among the systems under the study (Figure 1B). The results obtained from the interpeptide distance analysis were validated via an additional short replica for 250 ns of each system. Additional plots for each system under the study are available in the Supplementary Material (Figure S1). While quantitatively the values were different, qualitatively the trends remained the same.

**Figure 2:**
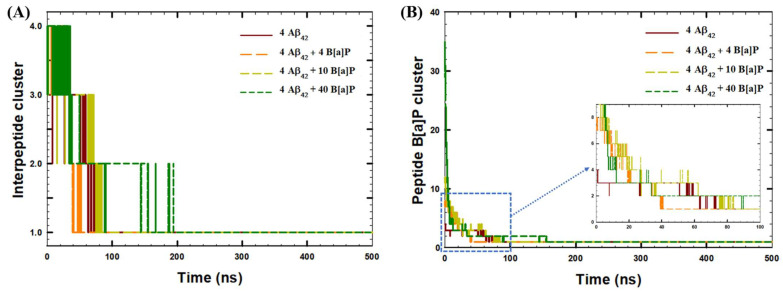
Time-evolution of (**A**) Formation of interpeptide clusters during 500 ns of the simulation, (**B**) formation of clusters of Aβ_42_ peptides and B[a]P molecules during 500 ns of the simulation.

With increasing the concentration of B[a]P molecules, i.e., using 10 and 40 molecules, the time required for tetramerization increased, and became 100 ns and 200 ns for the two systems, respectively. In addition, according to Figure 1C, the distance analyses between peptides and B[a]P molecules showed that B[a]P molecules were also bound to the peptides within 70 ns of the simulations in all systems under the study, indicating the formation of agglomerate encompassing the PAHs and the peptides. Furthermore, the aggregation kinetics of four Aβ_42_ peptides was analyzed in terms of the growth of the interpeptide clusters (Figure 2A), as well as the formation of the clusters of B[a]P and peptides (Figure 2B).

According to Figure 2A, the rate of tetramerization of four peptides was fastest in the presence of 4 B[a]P molecules (in ~52 ns of the MD run). In comparison, in the absence of B[a]P molecules, a stable single cluster of four peptides was produced in ~75 ns. In the presence of 10 B[a]P molecules, the cluster was formed in ~85 ns of the simulation, while in the presence of 40 B[a]P, formation of a stable cluster of four peptides was completed in ~200 ns of the production run. According to Figure 2B, the fastest formation of a stable peptide—B[a]P cluster occurred in ~40 ns of the simulation in the presence of 4 B[a]P molecules. In comparison, additional time was required for the complete aggregation of peptides and B[a]P molecules in the systems with 10 B[a]P molecules (~85 ns of the simulation) and 40 B[a]P molecules (~155 ns of the simulation).

**Figure 3:**
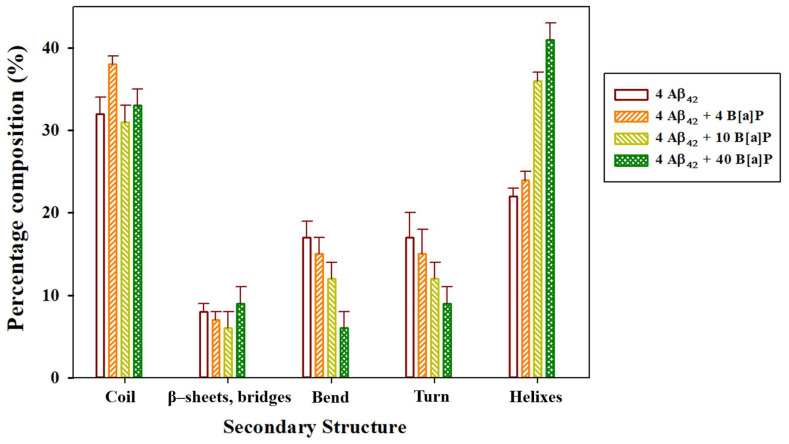
Composition of the secondary structure of the peptides averaged among the last 30 ns of the simulations.

**Figure 4:**
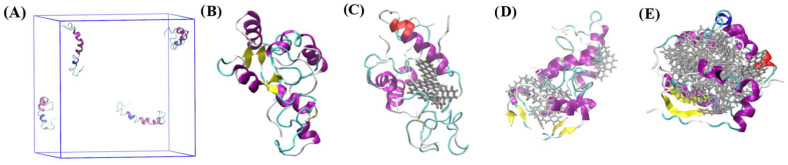
Representative snapshots of (**A**) four Aβ_42_ monomers before the simulation; (**B**) interpeptide cluster of four Aβ_42_ peptide monomers in the end of the simulation with no B[a]P; intermolecular cluster of four Aβ_42_ peptide monomers and B[a]P in the end of the simulations with (**C**) 4 B[a]P molecules; (**D**) 10 B[a]P molecules; (**E**) 40 B[a]P molecules. Color index: 1. Secondary structure: beta sheet = yellow, bridge − beta = tan, alpha helix = purple, 3_10_Helix = blue, Pi-Helix = red, turn, bend = cyan, coil = white, 2. B[a]P molecule = grey.

Overall, according to cluster analyses, the presence of 5.00 mM B[a]P enhanced the oligomerization kinetics, based on the time required to make one stable cluster of four Aβ_42_ peptide monomers, by 30%, in comparison to the aggregation kinetics of peptides in the absence of B[a]P. In contrast, the presence of 12.5 mM B[a]P and 50.0 mM B[a]P decreased the aggregation kinetics of peptides by 13% and 167%, respectively. In addition, in the system with 10 B[a]P molecules, the formation of interpeptide clusters and formation of peptide—B[a]P clusters occurred simultaneously, while in the systems with 4 and 40 B[a]P molecules, the formation of a stable interpeptide cluster occurred only after all B[a]P molecules were bound to peptides. The results indicated that oligomerization was essentially driven by the hydrophobic interactions between B[a]P and Aβ_42_ peptides [37], rather than the intrinsic interpeptide hydrophobic interactions [38]. The secondary structure of the interpeptide clusters was further analyzed for the last 30 ns of the simulations (Figure 3), wherein the tetramerized, clustered structure was stable. In addition, the intermolecular clusters observed at the end of the simulations, along with the initial monomer structure, were visualized via VMD (Figure 4).

The results of the secondary structure analysis obtained from our previous study [26] showed that in the presence of 1 B[a]P molecule, high amounts of the coils, bends, and β-content with low amount of helices were observed in the secondary structure of the peptide monomer. The results of this study (Figure 3) showed that the secondary structure of the oligomers would also depend on the concentration of the B[a]P molecules present in the simulation box. In particular, the analysis of the time-averaged secondary structure of Aβ_42_ peptides showed that in the presence of 4 B[a]P molecules, the coil region was predominant (38%). In comparison, in the presence of 10 and 40 B[a]P molecules, the helix region was prevalent (34% and 36%, respectively). Typically, the progression of Alzheimer’s disease is associated with the formation of β-sheet fibrils [39,40], however, more simulation time will be required for the formation of the stable β-sheets from the random coils in the peptide oligomers [41]. Interestingly, the β-content did not significantly vary with different B[a]P concentrations, with 6–9% of the β-content observed in all systems under the study. Nonetheless, the formation of the compact coil structures in Aβ oligomers is also an important step toward the formation of β-sheets [42]. In particular, in the presence of 4 B[a]P molecules, the high number of coils in the secondary structure of the peptides was correlated with enhanced oligomerization kinetics.

**Figure 5:**
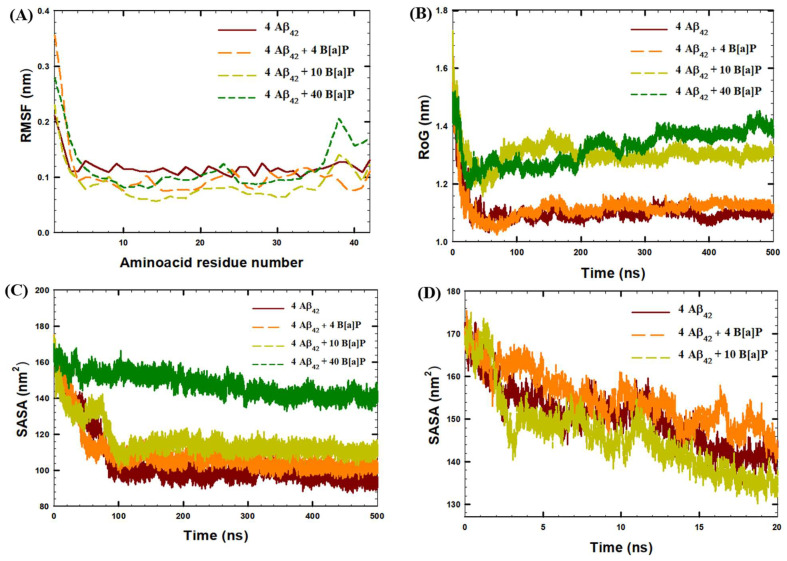
(**A**) RMSF of Aβ_42_ peptide residues, averaged among four peptides in the systems under the study, in the last 30 ns of the simulations, (**B**) Time-evolution of Radius of Gyration (RoG) of Aβ_42_ peptides, averaged among four peptides in the systems under the study, (**C**) Time-evolution of Solvent Accessible Surface Area (SASA) of Aβ_42_ peptides within 500 ns of the simulation, (**D**) Time-evolution of SASA of Aβ_42_ peptides within first 20 ns of the simulation in the systems with no B[a]P, with 4 B[a]P and 10 B[a]P molecules.

According to Figure 4A, in the beginning of the simulation, Aβ_42_ monomers consisted of helixes (Aβ_10–23_, Aβ_28–31_), turn, bend and coil regions (N- and C- terminuses), with no β—sheets observed in their secondary structures. At the end of 500 ns of the simulation system with no B[a]P had β-sheets in the regions of Aβ_31–36_ and Aβ_40_ of the C-terminus (Figure 4B). According to Figure 4C, in the presence of 4 B[a]P, β-sheets were observed in the regions of Aβ_28–32_ and Aβ_35–39_ at the end of the simulation. In the system with 10 B[a]P, the formation of the β-sheets in occurred in the regions of Aβ_2–5_ and Aβ_30–41_ (Figure 4D), whereas with 40 B[a]P, β-sheets were formed in the regions of Aβ_28–32_ and Aβ_35–39_ (Figure 4E). These observations showed that β-sheets were mainly formed in the regions of C-terminus, considered as a possible nucleation site for the self-aggregation, as was highlighted in the earlier literature [43,44]. In addition, in the presence of 4 B[a]P, the tetramerization occurred with the formation of oligomers with increased coil region, that could be expected to form β-sheets further [45]. In comparison, in the systems with 10 and 40 B[a]P molecules, the binding of numerous B[a]P to peptides via aromatic π-π interactions [46] resulted in the formation of B[a]P—peptide agglomerates. Consequently, typical oligomerization was suppressed due to the presence of numerous B[a]P molecules and their clusters, resulted in steric hindrance [47], as shown in Figure 4D,E. To investigate the deviations in the peptide structure with the formation of intermolecular clusters, RMSF, RoG, SASA, and H-bonds analyses were further performed (Figure 5, Table 2).

According to RMSF analyses of the last 30 ns of the simulations, the fluctuations in the positions of aminoacid residues in Aβ_42_ were in the range of ~0.1–0.22 nm, in the absence of B[a]P molecules (Figure 5A). In system 2 with 4 B[a]P molecules, in general, the RMSF values of the aminoacid residues were low in the Aβ_3–42_ segment (RMSF values of ~0.06–0.12 nm) with enhanced fluctuations in the N-terminus (RMSF values of ~0.36 nm in the Aβ_1–2_ region). In addition, the results showed that the presence of 4 B[a]P molecules stabilized the central hydrophobic core (RMSF values of ~0.09 nm in the Aβ_15–20_ region) and the beta-sheets region of the C-terminus (RMSF values of ~0.09 nm in the Aβ_26–28_ and Aβ_38–41_ regions), promoting the formation of tetramers. In the presence of 10 B[a]P molecules, the RMSF values of the aminoacid residues were low in Aβ_3–36_ segment (RMSF values of ~0.06–0.1 nm), indicating a similar effect of the tetramer stabilization. Moreover, in this system under study, the enhanced fluctuations were observed in the N- and C-terminuses, which were correlated with the suppression of oligomerization (RMSF values up to ~0.23 nm for Aβ_1–2_, and ~0.14 nm for Aβ_38–42_). Similarly, enhanced fluctuations in the positions of the amino acids of the N-terminus (Aβ_1–2_, RMSF values of ~0.28 nm), and C-terminus (Aβ_37–41_ segment RMSF values of ~0.14–0.21 nm) were observed in the system with 40 B[a]P molecules in the end of the simulation, indicating increased movement of the aminoacids in the peptide terminuses due to the interactions with large amounts of B[a]P molecules and the hydrophobic interactions with the PAHs, as was discussed previously.

This observation was further corroborated with the time-evolution of the radius of gyration (RoG) of the peptide. The RoG values, averaged between four Aβ_42_ peptides, decreased from the initial value of 1.6 nm to ~1.1 nm within the simulation time in the systems with no B[a]P and in the presence of 4 B[a]P molecules (Figure 5B). In contrast, in the presence of 10 B[a]P and 40 B[a]P molecules, the radius of gyration deviated more significantly, up to ~1.3 nm and ~1.4 nm, respectively, due to the interactions with large amounts of B[a]P molecules.

In agreement with cluster and intermolecular distance analyses, for the systems with no B[a]P molecules, with 4 B[a]P, and 10 B[a]P molecules, the SASA values significantly decreased within first 100 ns of the MD simulations, indicating the interpeptide binding with consequent loss of peptide surface area available for solvent (Figure 5C). In addition, according to Figure 5C and Table 2, the final values of SASA at 500 ns were comparatively high in the systems with high number of B[a]P molecules in the simulation box. In particular, in the end of the simulations, the SASA values of peptides were 94.6 nm^2^, 102.7 nm^2^, 110.0 nm^2^, and 139.2 nm^2^ in the systems with no B[a]P, with 4 B[a]P, 10 B[a]P, and 40 B[a]P molecules, respectively (Table 2). This observation indicates that binding of large amount of B[a]P to Aβ_42_ peptides would consequently inhibit the oligomerization. Moreover, it was also noted that in the first 20 ns of the simulations the aggregation of peptides was the fastest in the system with 10 B[a]P molecules (Figure 5D). However, the initial rate of aggregation of peptides, within 20 ns of the simulation, was facilitated in the presence of 10 B[a]P molecules, and the consequent aggregation of B[a]P molecules to peptides inhibited oligomerization of four peptides. The results obtained from the SASA analysis were validated via an additional short replica of 250 ns performed for all systems under the study. Additional plots for each system under the study are available in the Supplementary Material (Figure S2). While quantitatively the values were different, qualitatively the trends remained the same.

The analysis of H-bonds in the last 30 ns of the simulations showed increased amounts of H-bonds in the presence of 4 B[a]P molecules (119 ± 5 bonds), in comparison to the system with 40 B[a]P molecules (109 ± 5 bonds), indicating that the presence of a large amount of B[a]P molecules inhibited the formation of interpeptide H-bonds (Table 2). Overall, RoG, SASA, and H-bonds analyses showed that the presence of 10 and 40 B[a]P molecules could interfere with the oligomerization process by binding to Aβ_42_ peptides, leading to elevated radius of gyration, SASA of Aβ_42_ peptides, and suppressed interpeptide H-bonding.

**Figure 6:**
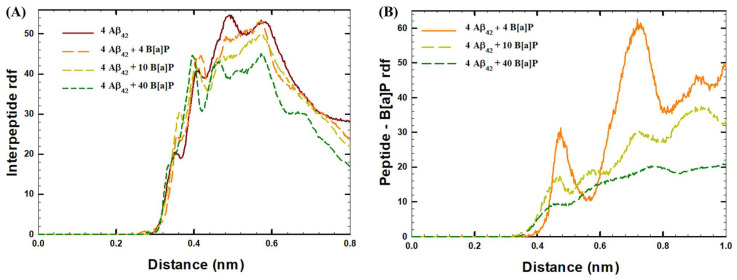
Radial distribution function (rdf) of (**A**) interpeptide interactions, (**B**) peptide—B[a]P ineractions in the systems under the study.

Furthermore, the radial distribution function (rdf) analysis showed that high interpeptide interactions were observed in the systems with no B[a]P molecules (maximum peaks of ~55 at 0.5 nm and ~53 at 0.6 nm on Figure 6A), and in the presence of 4 B[a]P molecules (maximum peak of ~53 at 0.6 nm on Figure 6A). In comparison, decreased interpeptide interactions in the presence of 10 B[a]P molecules (maximum peak value of ~50 at 0.6 nm on Figure 6A), and in the presence of 40 B[a]P molecules (highest peaks of ~45 at 0.4 nm and 0.6 nm on Figure 6A), indicated that interpeptide interactions were decreased with the addition of higher amounts of B[a]P molecules. According to the rdf analysis of peptide—B[a]P interactions (Figure 6B), strong peptide—B[a]P interactions were observed in the system with 4 B[a]P molecules (maximum peaks of ~30 at 0.47 nm and ~60 at 0.72 nm on Figure 6B). In comparison, low peptide—B[a]P interactions were observed in the systems with 10 B[a]P molecules (maximum peaks of ~17 at 0.47 nm and ~30 at 0.72 nm on Figure 6B) and 40 B[a]P molecules (maximum peaks of ~10 at 0.4 nm and ~20 at 0.76 nm on Figure 6B).

The energy analysis was further performed for the last 10 ns of the simulations to investigate the type of molecular interactions. According to Table 3, in the presence of B[a]P molecules, short-range electrostatic interpeptide interactions increased (Coul–SR), while short-range Lennard-Jones interactions (LJ–SR) between peptides decreased. These observations were also correlated to the enhanced hydrophobic interactions between peptides and B[a]P (LJ–SR), related to the aggregation of peptides and B[a]P molecules.

## 4. Conclusions

To conclude, molecular dynamics simulations revealed that with the addition of B[a]P molecules, electrostatic interactions between Aβ_42_ peptides increased due to the enhanced B[a]P—peptide hydrophobic interactions and binding of Aβ_42_ peptides and B[a]P. Although the presence of 4 B[a]P molecules did not significantly affect interpeptide H-bonding and interpeptide interactions, the presence of 10 and 40 B[a]P molecules suppressed H-bonding and decreased interpeptide interactions, observed in the end of the simulations. Overall, according to the results of our study, the presence of 5.00 mM B[a]P, i.e., 4 molecules, accelerated the formation of Aβ_42_ peptide tetramers by 30% and stabilized C-terminus of peptides, suggesting consequent progression of Alzheimer’s disease. Nonetheless, the effect of ultrafine air pollutants on AD would be more complicated due to the more complex composition of pollutants present in the environment.

## Figures and Tables

**Table 1:** Number of molecules in the simulated systems.

System	Aβ_42_	B[a]P	B[a]P Concentration	H_2_O	Na^+^	Cl^−^
4 Aβ_42_	4	0	0 mM	42,296	128	120
4 Aβ_42_ + 4 B[a]P	4	4	5.00 mM	42,270	128	120
4 Aβ_42_ + 10 B[a]P	4	10	12.5 mM	42,179	128	120
4 Aβ_42_ + 40 B[a]P	4	40	50.0 mM	41,798	128	120

**Table 2:** Solvent Accessible Surface Area (SASA) of peptides in the beginning (SASA_0 ns_), and in the end (SASA_500 ns_) of the simulation, minimum SASA (SASA_min_) values of peptides, and an average number of interpetide H—bonds observed in the last 30 ns of the simulation.

	SASA_0 ns_ (nm^2^)	SASA_500 ns_ (nm^2^)	SASA_min_ (nm^2^)	H-Bonds (Last 30 ns)
4 Aβ_42_	171.6	94.60	87.30	115 ± 5
4 Aβ_42_ + 4 B[a]P	169.0	102.7	95.10	119 ± 5
4 Aβ_42_ + 10 B[a]P	169.6	110.0	101.5	113 ± 5
4 Aβ_42_ + 40 B[a]P	166.5	139.2	132.7	109 ± 5

**Table 3:** Short-range (SR) and long-range (LR) Coulombic and Lennard-Jones potential between peptides, and peptide—B[a]P, observed in the last 10 ns of the simulations in the systems under the study.

System	Peptide–Peptide (kJ/mol)				Peptide–B[a]P (kJ/mol)	
	Coul–SR	LJ–SR	Coul–LR	LJ–LR	Coul–SR	LJ–SR
4 Aβ_42_	−44,048 ± 26	−3712 ± 11	27,804 ± 8	−38.5 ± 3.4	-	-
4 Aβ_42_ + 4 B[a]P	−44,333 ± 10	−3577 ± 7	28,052 ± 12	−44.1 ± 1.9	−49.2 ± 2.2	−423 ± 2
4 Aβ_42_ + 10 B[a]P	−43,963 ± 40	−3536 ± 9	28,042 ± 7	−7.9 ± 3	−60.6 ± 2.8	−762 ± 16
4 Aβ_42_ + 40 B[a]P	−43,945 ± 22	−3016 ± 7	28,043 ± 6	9.8 ± 3	−191.7 ± 3	−2020 ± 8

## Data Availability

The datasets generated and analyzed during the current study are available from the corresponding author on a reasonable request.

## Supplementary Materials

The following supporting information can be downloaded at: https://www.mdpi.com/article/10.3390/toxics10120786/s1, Figure S1: Time-evolution of average interpeptide distances in the systems with additional replicas; Figure S2: Time-evolution of Solvent Accessible Surface Area (SASA) of Aβ_42_ peptides in the systems with additional replicas.
