# Supplementary material for: Ambient Benzo[a]pyrene’s Effect on Kinetic Modulation of Amyloid Beta Peptide Aggregation: A Tentative Association between Ultrafine Particulate Matter and Alzheimer’s Disease
Source: Toxics. 2022 Dec 14;10(12):786. doi: 10.3390/toxics10120786 (PMC9785023; doi:10.3390/toxics10120786)
Supplement: Supplementary file 1 [file toxics-10-00786-s001.zip › toxics-2054091-supplementary.pdf]

Supplementary Material

"Ambient benzo[a]pyrene's effect on kinetic modulation of amyloid beta peptide aggregation: a tentative association between ultrafine particulate matter and Alzheimer's disease"

By Samal Kaumbekova, Mehdi Amouei Torkmahalleh, and Dhawal Shah

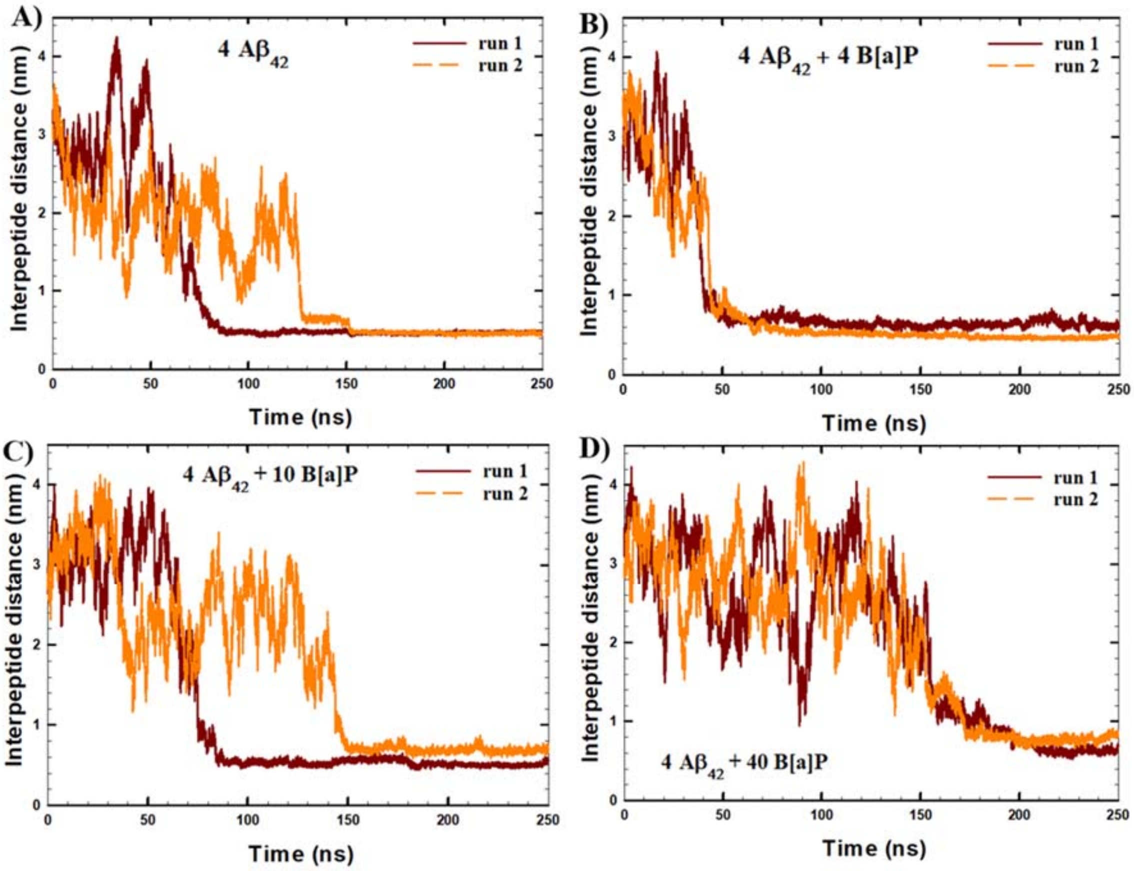

Figure S1. Time-evolution of average interpeptide distances in the systems with additional replicas: A) 4 $A\beta_{42}$ peptides, B) 4 $A\beta_{42}$ peptides and 4 B[a]P, C) 4 $A\beta_{42}$ peptides and 10 B[a]P, D) 4 $A\beta_{42}$ peptides and 40 B[a]P.

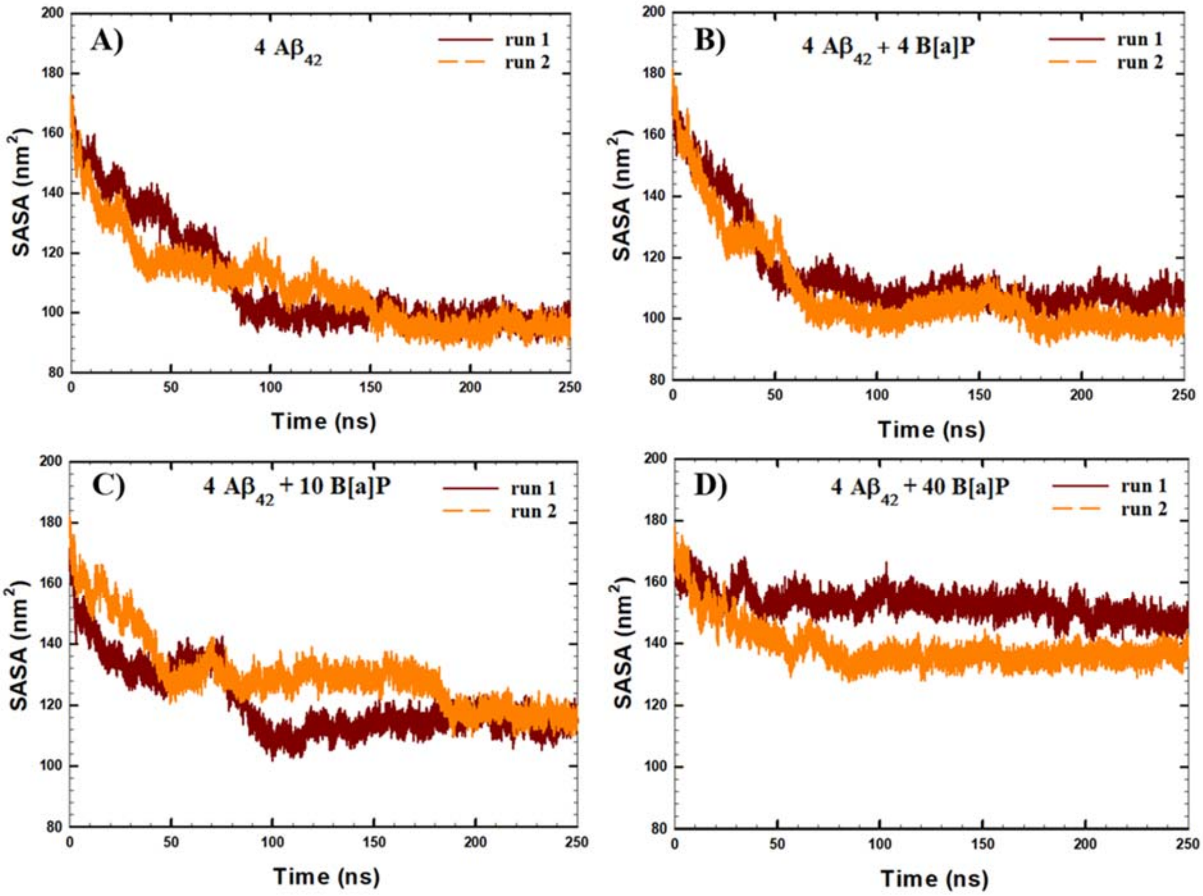

Figure S2. Time-evolution of Solvent Accessible Surface Area (SASA) of $A\beta_{42}$ peptides in the systems with additional replicas: A) 4 $A\beta_{42}$ peptides, B) 4 $A\beta_{42}$ peptides and 4 B[a]P, C) 4 $A\beta_{42}$ peptides and 10 B[a]P, D) 4 $A\beta_{42}$ peptides and 40 B[a]P.
